# Local LLM-based sentiment analysis of emotional patterns in complex-to-manage and treatment-resistant psoriatic arthritis

**DOI:** 10.1007/s11739-026-04396-w

**Published:** 2026-06-07

**Authors:** Vincenzo Venerito, Sergio Del Vescovo, M. Cameron Hay, Shikha Singla, Christine Lindsay, Philip Judson Mease, Fabian Proft, Andre Lucas Ribeiro

**Affiliations:** 1 Rheumatology Unit, DiMePre-J, University of Bari “Aldo Moro”, Bari, Italy; 2 Department of Anthropology, Miami University, Oxford, OH USA; 3 Department of Rheumatology, Medical College of Wisconsin, Milwaukee, WI USA; 4 GRAPPA Patient Research Partner, Prosper, TX USA; 5 Department of Rheumatology, Providence Swedish Medical Center and University of Washington, Seattle, WA USA; 6 Department of Gastroenterology, Infectiology and Rheumatology (Including Nutrition Medicine), CharitéUniversitätsmedizin Berlin, corporate member of Freie Universität Berlin and Humboldt-Universität zu Berlin, Berlin, Germany; 7 Rheumatology Division at Hospital de Clínicas de Porto Alegre, Porto Alegre, Brazil; 8 Rheumatology Division at Hospital Moinhos de Vento, Porto Alegre, Brazil

**Keywords:** Psoriatic arthritis, Patient narratives, Sentiment analysis, Emotional patterns, Large language models, Difficult to treat

## Abstract

Psoriatic arthritis (PsA) presents significant challenges, with many patients experiencing inadequate treatment response. While clinical aspects of suboptimal disease control have been characterized, the emotional burden of treatment failure remains poorly understood. Our objective was to systematically characterize the emotional patterns expressed by PsA patients when describing treatment failures and management challenges. A multi-national online survey with PsA patients was conducted through GRAPPA (Group for Research and Assessment of Psoriasis and Psoriatic Arthritis). The survey was conducted to explore their perspectives on how to define complex-to-manage (C2M)-PsA and treatment-refractory (TR-) PsA, and how these conditions impact their lives. Open-ended responses were analyzed using a locally deployed Meta Llama 3.2 3B Instruct large language model, applying Ekman’s six basic emotion framework and the valence-arousal dimensional model. Sentiment analysis revealed predominantly negative emotional patterns (mean valence −0.57 ± 0.25) with moderate-to-high arousal (0.65 ± 0.10). Sadness was most prevalent (54.27%), followed by fear (43.59%). Disease duration (≥ 5 years) showed similar valence scores to shorter durations. The number of reported challenging disease aspects negatively correlated with valence (*r* = −0.16, *p* = 0.02) and positively with fear (*r* = 0.15, *p* = 0.02). Axial involvement and comorbidities were significantly associated with fear as the dominant emotion. Hierarchical clustering identified four distinct emotional profiles, with high multi-domain burden clusters showing the most negative valence. Natural language processing-based sentiment analysis characterizes emotional patterns in PsA patient narratives. Negative emotions, particularly sadness and fear, predominated, with specific disease features linked to these emotional states, underscoring the need for approaches addressing both physical and emotional dimensions in PsA.

**Supplementary Information:**

The online version contains supplementary material available at https://doi.org/10.1007/s11739-026-04396-w.

## Introduction

The complex nature of psoriatic arthritis (PsA) and its multifaceted impact on patients have led to the recognition of Complex-to-Manage PsA (C2M-PsA) and Treatment-Refractory PsA (TR-PsA), both characterized by varying degrees of treatment ineffectiveness. TR-PsA is defined by objective evidence of persistently active inflammation despite appropriate therapy, whereas C2M-PsA encompasses all potential reasons for contributing to treatment failure, including but not limited to TR-PsA [1, 2]. Despite advances in therapeutic options [3], a significant proportion of patients continue to experience suboptimal disease control and repeated therapeutic failures [4].

Although considerable attention has been given to the clinical and therapeutic aspects of treatment failure, the emotional burden associated with C2M- and TR-PsA remains incompletely characterized. This gap is clinically relevant, as emotional well-being has been shown to significantly influence treatment adherence, therapeutic outcomes, and overall quality of life in chronic inflammatory diseases [5–8]. Moreover, recent studies have highlighted significant discordance between patient and clinician priorities in PsA management—with patients prioritizing concerns about future health, uncertainty, and access to care—issues that are often underrecognized during routine clinical encounters [9]. Together, these findings underscore the need for systematic approaches to capture the emotional dimensions of treatment challenges in PsA.

In this regard, recent advances in natural language processing (NLP) and artificial intelligence [10, 11] have created new opportunities to analyze and interpret patient-reported experiences in a more nuanced and comprehensive manner [12–14]. Large language models (LLMs), in particular, offer the potential to systematically analyze emotional content in patients’ narratives, providing insights that may not be readily apparent through traditional quantitative assessments [15].

Established frameworks from affective science provide a robust theoretical foundation for the assessment of emotional patterns in patient narratives. The dimensional model of emotion, particularly Russell’s valence-arousal framework, quantifies affective states along two primary dimensions: valence, which ranges from pleasant to unpleasant emotions, and arousal, which captures the intensity of emotional activation, from calm to excited states [16]. Complementing this dimensional approach, Ekman’s discrete emotion theory identifies six basic emotional categories (i.e., anger, disgust, fear, joy, sadness, and surprise), which are considered universal across cultures and fundamental to human experience [17, 18]. Integrating these two frameworks allows for a richer characterization of affect, combining categorical classifications with continuous metrics to capture subtleties that may not align neatly with a single emotional label.

Within this conceptual framework, the present study applied NLP techniques to identify and quantify emotional patterns embedded in PsA patients responses. We implemented a locally deployed LLM for sentiment analysis, a process that identifies and quantifies affective states and subjective information from text, to evaluate the affective dimensions of patient-reported experiences related to treatment challenges and their associations with clinical features. Moreover, we identified distinct patient subgroups based on disease burden profiles and analyzed their associated emotional patterns.

Our objective was to systematically characterize the emotional patterns expressed by PsA patients when describing treatment failures and management challenges. While textual emotional expression does not directly equate to explicit psychological needs, understanding these affective patterns could provide clinicians with additional insights into the experiences of patients facing refractory disease, potentially informing more comprehensive approaches to patient care.

## Methods

### Study design and participants

An international online survey was conducted through GRAPPA (Group for Research and Assessment of Psoriasis and Psoriatic Arthritis), with the goal of exploring patient perspectives on how to define TR-PsA and C2M-PsA and how these conditions impact their lives. The development of the survey was guided by collaborative discussion among GRAPPA members and patient research partners (PRPs); the questionnaire was then translated and made available in ten different languages to enhance accessibility. A rigorous translation process was employed, where each version of the survey was initially translated with DeepL and then reviewed by two native speakers and by a PRP fluent in the respective language. Participation was voluntary, with no financial incentives offered, and respondents had the option to skip any question or withdraw from the survey at any time to ensure their autonomy; moreover, to maintain confidentiality, responses were collected anonymously.

The final survey was structured into three main sections: demographic data, structured questions regarding reported disease characteristics, and four open-ended questions. These were designed to elicit personal definitions of TR-PsA (Q1) and C2M-PsA concepts (Q2), their life impacts (Q3), and additional patient perspectives (Q4). Notably, among the structured questions, patients were asked to identify challenging disease aspects from a list of 12 manifestations that made their condition "difficult to treat/complex to manage".

The study was approved by the independent ethics committee of the Charité Universitätsmedizin Berlin, Germany (EA1/225/23) and conducted in accordance with the 1964 Declaration of Helsinki and its later amendments.

### Sentiment analysis methodology

Due to the qualitative nature of the open-ended responses, as an exploratory approach, a computational sentiment-analysis was conducted to systematically analyze and categorize these narrative data. Prior to sentiment analysis, all non-English responses were translated into English using DeepL, consistent with the translation approach adopted for the survey itself. Translated texts were then processed by the LLM identically to English-language responses. The analysis was performed using an LLM locally deployed on a MacBook Pro M4 Ultra with 128 GB RAM, namely Meta Llama 3.2 3B Instruct, which was specifically prompted (see Supplementary File 1) to assess emotions based on two frameworks: Ekman’s six basic emotions theory (anger, disgust, fear, joy, sadness, surprise) and the valence-arousal dimensional model.

The prompting strategy employed an instruction-style sentiment analysis methodology, which has been demonstrated to be effective when annotation resources are limited [19]. This approach involves providing the LLM with a small number of representative examples for each emotion category, enabling the model to better understand the task requirements and emotional patterns specific to patient responses. Moreover**,** it allowed us to provide healthcare-relevant examples (e.g., "The doctors don’t listen to me" for anger, "I can’t function anymore" for sadness), enabling the model to better recognize emotional expressions within the medical context. Moreover, the LLM was configured with a temperature parameter of 0.3 to balance response consistency with contextual sensitivity.

The model was instructed to return a percentage distribution across Ekman’s six basic emotions, with normalization applied to guarantee that emotion percentages summed to exactly 100% for each patient response. In parallel, emotional dimensions were quantified using valence (emotional positivity/negativity on a + 1 to −1 scale) and arousal (physiological activation on a 0–1 scale).

The dominant emotion was defined as the emotion with the highest percentage for that response. Moreover, only for the patients who provided complete responses to Q1, Q2 and Q3, the mean values of emotional dimensions across these questions were calculated in order to create a comprehensive emotional profile (Q4 was excluded due to the high rate of missing answers, *n* = 365/570, 64.04%). Then, the Q1–Q3 mean value for each patient was used for all subsequent analyses. Of note, duplicate responses across questions were not frequent and were included in the analysis, as patients who chose to repeat responses possibly intended to emphasize their perspective, with each question analyzed independently to maintain analytical consistency.

### Human annotation and reliability.

To validate automated sentiment outputs, we manually annotated a gold-standard subset comprising 20% of analyzable full responses to ensure representativeness. Two board-certified rheumatologists, blinded to all LLM outputs and patient characteristics, independently assigned the dominant Ekman emotion (anger, disgust, fear, joy, sadness, or surprise) to each text after a short calibration session using six exemplar cases. Inter-rater reliability was quantified using Cohen’s κ with 95% confidence intervals (CI). Discrepancies were resolved by consensus to create the human-gold labels, which were subsequently compared with the LLM-generated classifications.

### Statistical analyses

All statistical analyses were performed using JMP Pro version 17.0®. Summary statistics were calculated for demographic variables, reported challenging disease aspects, and emotional dimensions. Disease duration was dichotomized as shorter (< 5 years) or longer (≥ 5 years) as used in recent PsA clinical trial programs [20, 21] and to prevent class imbalance between disease duration subgroups.

T-test, Chi-square test and analysis of variance (ANOVA) were conducted for comparisons among different patient groups. Pearson correlation coefficients were calculated to assess bivariate associations between emotional dimensions and reported challenging disease aspects. Linear regression and logistic regression models evaluated relationships between disease aspects and emotional dimensions. Hierarchical cluster analysis (Ward’s method) was performed on standardized variables representing challenging disease aspects only. Valence, arousal, and Ekman’s emotion distributions were subsequently compared across clusters as independent outcomes to characterize the emotional patterns associated with each clinical profile. Then, a comparison of emotional patterns between the different clusters was conducted. We assessed assumptions for parametric tests; where violated, we used non‑parametric alternatives (Mann–Whitney U, Kruskal–Wallis). We controlled the false discovery rate using Benjamini–Hochberg within families of related tests. We reported effect sizes (ORs) and 95% CIs alongside p‑values (two‑sided, *α* = 0.05). Logistic models for dominant emotion (fear vs sadness) prespecified covariates (age, sex, language, disease duration, PsA clinical domains, comorbidities).

## Results

### Sentiment analysis

The survey included 570 PsA patients, predominantly female (68.8%), with most aged 50–59 years (32.3%) or ≥ 60 years (27.4%). The majority were White (72.6%), married/partnered (65.6%), had secondary education (65.6%), and were employed full-time (35.8%) or retired (20.4%). Mean disease duration (± SD) was 14.43 ± 11.59 years. Among the predefined disease aspects, arthritis, fatigue, and skin psoriasis were the most commonly reported by patients as contributing to their condition being TR and/or C2M. Of the 570 respondents, 234 (41.1%) provided complete responses to Q1, Q2, and Q3 and were included in the sentiment analysis. The sociodemographic characteristics of this analyzed subset are reported in Supplementary Table 1 alongside the total cohort; notably, the subset included a higher proportion of females and showed a different educational distribution, with a greater representation of college- and postgraduate-educated respondents. Disease characteristics were broadly comparable between the two groups [Supplementary Table 2].

**Fig. 1:**
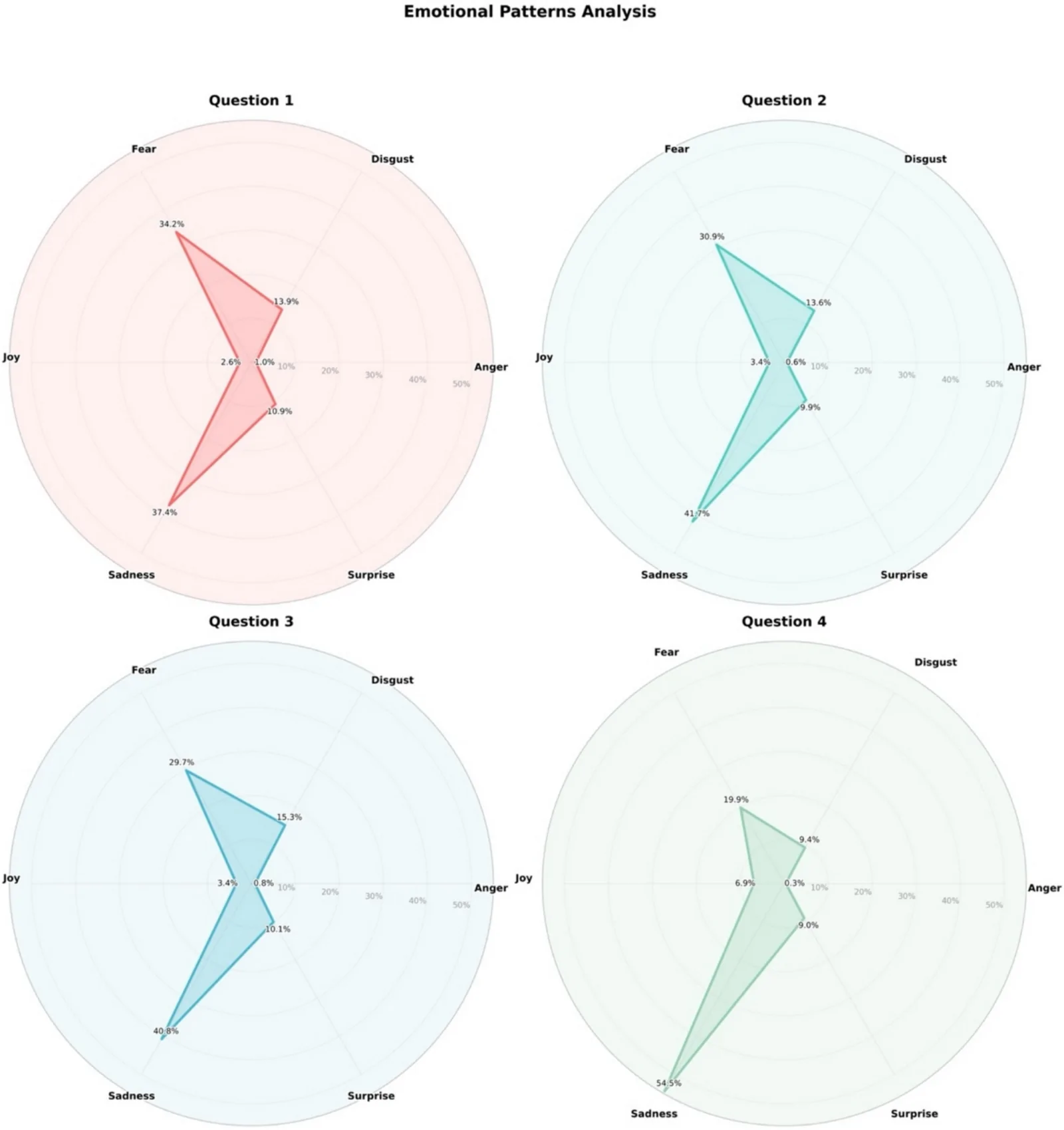
Distribution of Ekman’s six basic emotions (anger, disgust, fear, joy, sadness, and surprise) across the four open-ended survey questions (Q1–Q4). For each question, bars represent the mean percentage allocation of each emotion across all respondents who provided a response to that question. Q1 and Q2 asked patients to define treatment-refractory (TR-PsA) and complex-to-manage PsA (C2M-PsA), respectively; Q3 explored the impact of these conditions on daily life; Q4 invited any additional patient perspectives

The sentiment analysis, conducted entirely by the LLM on 234 patients who provided complete responses to questions Q1, Q2, and Q3, revealed predominantly negative emotional patterns across all questions [Fig. 1, Table 1]. The overall mean valence was −0.57 ± 0.25, indicating a negative emotional tone, while arousal levels were moderate to high (0.65 ± 0.10). Among the discrete emotions, sadness was the most prevalent (38.80 ± 14.17%), followed by fear (32.73 ± 11.38%) and disgust (14.25 ± 6.56%). Surprise accounted for 10.38 ± 4.77%, whereas joy (2.98 ± 3.13%) and anger (0.86 ± 2.71%) were notably infrequent. Overall, sadness emerged as the dominant emotion in 127 patients (54.27%), followed by fear in 102 patients (43.59%), and disgust in only 5 patients (2.13%).

**Table 1:** Emotional responses for each open‑ended question (*Q*1–*Q*4) and the aggregate *Q*1–*Q*3 profile. Values are means (± SD). Entries for Ekman emotions (anger, disgust, fear, joy, sadness, surprise) are the means of per‑response percentage allocations (%), with emotion percentages normalized to sum to 100% for each response. The mean percentage values represent the average proportional intensity of each emotion across participants, whereas the dominant emotion frequencies (in “Results”) reflect the proportion of individuals for whom that emotion had the highest relative weight. Consequently, the overall means and dominance frequencies are not expected to coincide exactly. Valence ranges from − 1 (most negative) to + 1 (most positive); arousal ranges from 0 (low activation) to 1 (high). The aggregate *Q*1–*Q*3 column includes only patients with complete *Q*1–*Q*3 responses (*n* = 234) and was used for all inferential analyses (correlations, regressions, clustering). *Q*4 is presented for descriptive completeness only and was excluded from inferential analyses due to substantial missing/brief responses (*n* = 106). See Supplementary Tables for cohort characteristics and subgroup summaries

	*Q*1, mean (SD)	*Q*2, mean (SD)	*Q*3, mean (SD)	Aggregate *Q*1–*Q*3, mean (SD)	*Q*4, mean (SD)
Valence	−0.67 (0.35)	−0.41 (0.48)	−0.57 (0.48)	−0.57 (0.25)	−0.02 (0.67)
Arousal	0.68 (0.15)	0.63 (0.17)	0.63 (0.17)	0.65 (0.10)	0.61 (0.17)
Anger	1.03 (4.35)	0.61 (3.68)	0.81 (5.13)	0.86 (2.71)	0.34 (2.42)
Disgust	13.87 (9.67)	13.56 (12.12)	15.31 (13.41)	14.25 (6.56)	9.40 (11.26)
Fear	34.17 (18.55)	30.89 (19.31)	29.65 (19.95)	32.73 (11.38)	19.90 (19.59)
Joy	2.62 (5.21)	3.39 (6.75)	3.38 (8.07)	2.98 (3.13)	6.87 (12.56)
Sadness	37.42 (21.75)	41.69 (24.35)	40.79 (24.48)	38.80 (14.17)	54.46 (28.09)
Surprise	10.91 (8.15)	9.86 (8.41)	10.07 (9.01)	10.38 (4.77)	9.03 (9.13)

In the human-annotated subset (47/234, 20.1%), inter-rater agreement between the two rheumatologists was substantial to excellent, with Cohen’s *κ* = 0.81 (95% CI 0.74–0.87). Agreement between the LLM output and the human–gold consensus was similarly high (*κ* = 0.78 [95% CI 0.70–0.85]), supporting good validity of the automated emotion annotations.

The emotional responses, as measured by mean sentiment scores across Q1–Q3, showed consistent patterns across demographic subgroups. Hence, analysis revealed no significant differences in valence and arousal scores when comparing across different genders, age groups, languages spoken, ethnicities, educational levels, marital statuses, and occupational statuses (Supplementary Table 3). For Ekman’s emotions, the majority of subgroup comparisons were also non-significant, with only isolated differences observed for the emotions joy and surprise in specific demographic categories (Supplementary Table 4).

### Emotions and disease features

Notably, no significant differences were found between patients with longer disease duration (*n*. 144/234, 61.54%) and those with shorter duration (n. 90/234, 38.46%) in either valence scores (≥ 5 years: −0.56 ± 0.27 vs < 5 years: −0.56 ± 0.21, *p* = 0.99) or arousal scores (≥ 5 years: 0.65 ± 0.09 vs < 5 years: 0.64 ± 0.10, *p* = 0.58), indicating similar emotional states regardless of disease duration. Among patients with a shorter disease duration, sadness was the dominant emotional tone in 56% compared to 54% in the long-duration group, while fear was the dominant emotion in 44% versus 43.2% in the long-duration group (*p* = 0.30).

The analysis of challenging disease aspects reported by the patients revealed significant correlations with emotional dimensions. The number of reported challenging aspects showed a negative correlation with valence (*r* = −0.16, *p* = 0.02), and a positive correlation with fear (*r* = 0.15, *p* = 0.02). Also, linear regression analysis revealed that the number of challenging disease aspects significantly influenced valence scores (*β* = −0.02, *p* = 0.01, 95% CI: − 0.031 to − 0.004). Thus, an increased number of reported challenging disease aspects were associated with more negative valence values. No significant relationship was found between this count and arousal scores.

Also, we examined associations between specific PsA features and patients’ dominant emotions. For this analysis, we dichotomized the emotional responses into "Sadness" versus "Fear" emotions to address the statistical limitations of low cell counts for the other dominant emotion (disgust).

Notably, among patients who reported axial disease as a challenging aspect of their disease, sadness was reported as the dominant emotion in 47.8% compared with 63.5% in those without axial involvement (*p* = 0.02), while fear was reported as dominant in 49.6% versus 34.4%, respectively (*p* = 0.02). Similarly, in the presence of other comorbidities (including obesity, hypertension, osteoporosis, and diabetes), sadness was the dominant emotion in 46.9% of cases compared with 58.6% among those without comorbidities (*p* = 0.04), whereas fear was dominant in 51.8% versus 38.8%, respectively (*p* = 0.04). On the other hand, no associations were observed for arthritis, enthesitis, dactylitis, psoriasis, nail involvement, uveitis, depression/anxiety, chronic pain, or fatigue (all *p* > 0.05). These differences were confirmed through a logistic regression model, where axial disease (OR = 1.87, 95% CI 1.09–3.22; *p* = 0.02) and other comorbidities (OR = 1.77, 95% CI 1.02–3.07; *p* = 0.04) showed significant associations with fear as the dominant emotion (fear vs sadness).

Further analysis of emotional dimensions revealed associations between specific challenging disease aspects and valence scores. Patients who reported enthesitis as a challenging aspect of their disease showed more negative valence scores compared to those who did not (−0.60 ± 0.24 vs −0.52 ± 0.26, *p* = 0.01). Similarly, patients who reported other comorbidities (obesity, hypertension, osteoporosis, diabetes) as challenging demonstrated more negative valence scores (−0.62 ± 0.23 vs −0.53 ± 0.26, *p* = 0.006). For arousal scores, only chronic pain showed a significant association, with patients reporting this as challenging demonstrating higher arousal levels (0.66 ± 0.09 vs 0.63 ± 0.10, *p* = 0.04).

### Cluster analysis

**Fig. 2:**
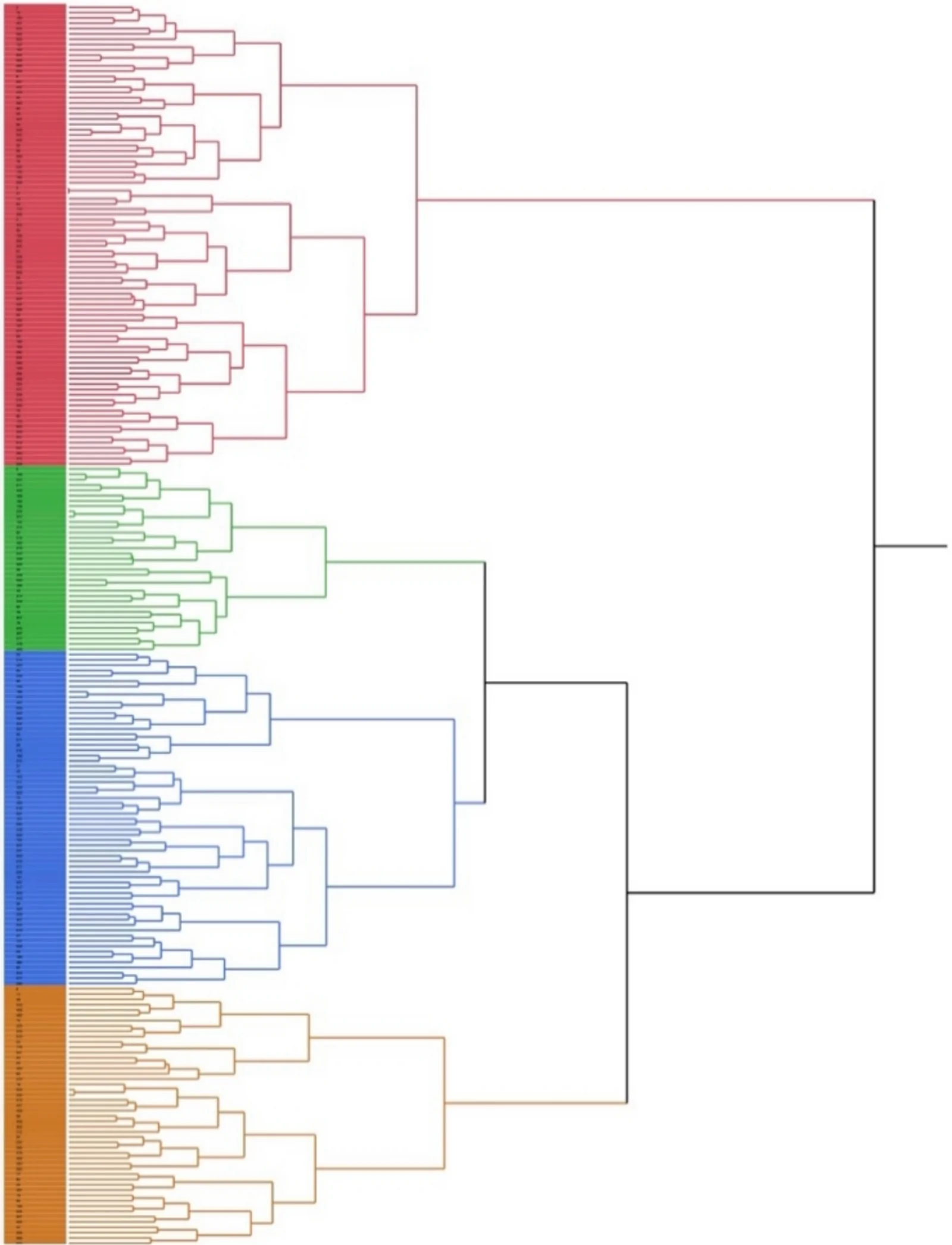
Hierarchical clustering with Ward’s method. Clustering was performed after column standardization of responses to questions about disease aspects making their condition difficult to treat and/or complex to manage. Each row represents a patient: the analysis identified four distinct patient clusters. The dendrogram shows relationships between clusters

Hierarchical cluster analysis identified four distinct patient groups [Fig. 2]: a "high multi-domain burden" cluster (Cluster 1, *n* = 87), characterized by the high rates of arthritis (82.76%), enthesitis (60.92%), dactylitis (49.43%), fatigue (85.06%), and chronic pain (66.67%) as a challenging disease; an "axial-predominant" cluster (Cluster 2, *n* = 35), with the highest prevalence of axial disease (82.86%) and moderate levels of other manifestations; a "skin-predominant burden" cluster (Cluster 3, *n* = 63), distinguished by the highest rates of skin psoriasis (71.43%), nail involvement (57.14%), depression/anxiety (52.38%), and axial disease (69.84%); and a "low burden with high fatigue" cluster (Cluster 4, *n* = 49), which showed lower prevalence across most disease manifestations but notably high fatigue levels (85.71%).

Emotional patterns differed significantly across clusters. Valence scores varied significantly (*p* < 0.001), with Cluster 1 showing the most negative valence (−0.74 ± 0.12), progressively less negative in Cluster 2 (−0.67 ± 0.12) and Cluster 3 (−0.52 ± 0.18), and least negative in Cluster 4 (−0.20 ± 0.18). Arousal levels also differed significantly (*p* < 0.001), with Cluster 1 showing the highest arousal (0.72 ± 0.06), while Cluster 2 showed the lowest levels (0.55 ± 0.07). Fear varied significantly across clusters (p < 0.001), with Clusters 1, 2, and 3 showing similar elevated levels (35.13 ± 10.89, 33.24 ± 10.58, and 33.73 ± 9.94, respectively), while Cluster 4 showed notably lower fear (26.94 ± 12.72). Sadness differed significantly (p < 0.001), with Cluster 4 showing the highest levels (47.21 ± 16.15) compared to the other clusters (range: 36.10–36.92). Joy also varied significantly (*p* = 0.01), increasing progressively from Cluster 2 (2.00 ± 1.67) to Cluster 4 (4.16 ± 3.84). Disgust also differed among the clusters (*p* = 0.03), with Cluster 2 exhibiting the highest levels (16.60 ± 8.90) and Cluster 4 the lowest (12.23 ± 6.02) [Table 2].

**Table 2:** Sentiment analysis results and reported challenging disease aspects across the four patient clusters in difficult-to-treat psoriatic arthritis. Values are expressed as mean (± SD) for sentiment analysis variables and *n* (%) for challenging disease aspects. *P*-values refer to global group differences across clusters (ANOVA or *χ*^2^ test as appropriate). *P*-values were adjusted for multiple testing using the Benjamini–Hochberg false-discovery-rate procedure (*α* = 0.05); adjusted values are reported in the rightmost column

	Cluster 1 (*n* = 87)	Cluster 2 (*n = *35)	Cluster 3 (*n* = 63)	Cluster 4 (*n* = 49)	*p*-value	BH adjusted *p*-value
**Sentiment analysis, mean (SD)**						
Valence	−0.74 (0.12)	−0.67 (0.12)	−0.52 (0.18)	−0.20 (0.18)	< 0.001	0.020
Arousal	0.72 (0.06)	0.55 (0.07)	0.63 (0.06)	0.59 (0.10)	< 0.001	0.010
Anger	1.27 (2.98)	1.22 (4.15)	0.66 (2.07)	0.14 (0.95)	0.09	0.095
Disgust	14.76 (5.74)	16.60 (8.90)	14.25 (6.72)	12.23 (6.02)	0.03	0.050
Fear	35.13 (10.89)	33.24 (10.58)	33.73 (9.94)	26.94 (12.72)	< 0.001	0.007
Joy	2.67 (2.46)	2.00 (1.67)	2.93 (3.70)	4.16 (3.84)	0.01	0.018
Sadness	36.20 (13.41)	36.10 (11.10)	36.92 (13.09)	47.21 (16.15)	< 0.001	0.005
Surprise	9.97 (4.62)	10.85 (4.93)	11.53 (4.87)	9.33 (4.66)	0.07	0.082
**Challenging disease aspects, *n* (%)**						
Arthritis	72 (82.76)	25 (71.43)	40 (63.49)	34 (69.39)	0.05	0.067
Enthesitis	53 (60.92)	22 (62.86)	37 (58.73)	18 (36.73)	0.03	0.046
Dactylitis	43 (49.43)	11 (31.43)	17 (26.98)	16 (32.65)	0.03	0.043
Skin psoriasis	45 (51.72)	5 (14.29)	45 (71.43)	21 (42.86)	< 0.001	0.004
Nails	31 (35.63)	1 (2.86)	36 (57.14)	11 (22.45)	< 0.001	0.003
Axial disease	44 (50.57)	29 (82.86)	44 (69.84)	22 (44.90)	< 0.001	0.003
Uveitis	14 (16.09)	1 (2.86)	12 (19.05)	8 (16.33)	0.08	0.089
IBD	11 (12.64)	6 (17.14)	6 (9.52)	5 (10.20)	0.71	0.710
Depression/anxiety	33 (37.93)	5 (14.29)	33 (52.38)	13 (26.53)	< 0.001	0.003
Chronic pain	58 (66.67)	18 (51.43)	29 (46.03)	25 (51.02)	0.06	0.075
Fatigue	74 (85.06)	27 (77.14)	38 (60.32)	42 (85.71)	0.002	0.004
Other comorbidities	34 (39.08)	10 (28.57)	31 (49.21)	8 (16.33)	0.002	0.004
Disease duration, years, mean (SD)	16.42 (12.61)	13.08 (7.70)	15.49 (12.87)	15.04 (10.28)	0.54	0.567

## Discussion

This exploratory sentiment analysis of GRAPPA’s patient survey examined the emotional perspectives of individuals with PsA regarding treatment refractoriness and their understanding of the concepts of TR- and C2M-PsA. By applying NLP to open-ended responses, we sought to capture the emotional dimension of patient narratives—a component often underrepresented in conventional clinical assessments.

Our analytical approach integrated two complementary theoretical frameworks from affective science: Ekman’s six basic emotions theory and the dimensional valence-arousal model. These models were selected to capture both discrete emotional categories and continuous affective dimensions. Although Ekman’s model has been debated for its methodological limitations [22], its operational clarity and widespread use make it particularly suitable for computational text analysis. Importantly, our goal was not to assert the universality of these emotional constructs, but rather to apply a structured and interpretable framework for categorizing emotional content in narrative data.

It is noteworthy that four of the six basic emotions in Ekman’s model are traditionally categorized as “negative” (i.e., anger, disgust, fear, and sadness), a distribution consistent with the expected emotional tone of responses concerning treatment failure. Similarly, the valence-arousal framework quantifies affective states along continuous dimensions of valence (−1 = most unpleasant to + 1 = most pleasant) and arousal (0 = low to 1 = high activation) [16]. Lower valence values, therefore, indicate more negative emotional experiences, while higher scores reflect more positive emotional states.

To our knowledge, this is the first application of a locally deployed LLM to analyze emotional patterns in PsA patients’ responses to open-ended questions about treatment refractoriness and its impact on daily life. The survey design intentionally included separate items on TR- and C2M-PsA (Q1 and Q2) to acknowledge distinctions between these overlapping concepts. Nevertheless, the emotional patterns observed in responses to both concepts were similar, suggesting comparable emotional expression regardless of the specific terminology. Sentiment analysis revealed consistently negative valence scores with moderate arousal levels across Q1–Q3.

Within Ekman’s framework, negative emotions predominated, with sadness and fear emerging as the primary emotions in 54.27% and 43.59% of patients, respectively. Sadness, characterized by withdrawal, introspection, and cognitive focus on loss and disappointment [23, 24], likely reflects both the inherent grief of chronic disease and the discouragement secondary to multiple treatment failures. Moreover, it is well established in the literature that PsA patients show increased rates of depression compared to both the general population and those with psoriasis alone [25–27]. Although depression and sadness are not synonymous, the psychological profile of sadness—diminished motivation, helplessness, and reduced pleasure—mirrors the psychosocial burden of PsA, including poor sleep, fatigue, and negative body image. In this survey, sadness may thus reflect the disappointment with failed therapies as well as loss of confidence in medical interventions.

Fear, the second most dominant emotion, is marked by heightened arousal, hypervigilance, and anticipatory worry about potential threats [28, 29]. This aligns with evidence of increased anxiety among PsA patients [30], where fear may focus on disease progression, treatment failure or social rejection. Its anticipatory nature may be especially relevant in treatment-resistant contexts, where uncertainty about future therapeutic options and disease trajectory predominates. As expected, joy was infrequently expressed—consistent with the survey’s focus on treatment challenges and the chronic burden of PsA.

Valence, arousal, and mean sentiment scores were largely consistent across demographic subgroups, suggesting that emotional signatures associated with treatment challenges transcend individual characteristics such as gender, age, ethnicities, language spoken, educational levels, marital status, and occupational status (Supplementary Table 3, Supplementary Table 4). Similarly, disease duration did not influence emotional responses, as patients with longer disease duration (≥ 5 years) demonstrated similar valence and arousal scores compared to those with shorter duration. The persistence of similarly negative emotions underscores the chronic psychological burden associated with treatment-resistant PsA. The observed correlation between the number of challenging disease aspects and more negative valence further provides quantitative evidence of the cumulative emotional impact of PsA, reinforcing its conceptualization as a complex, multi-domain disease in which psychological distress scales with clinical burden.

Analysis of specific disease aspects yielded additional insights. Patients reporting axial disease or comorbidities (e.g., obesity, hypertension, osteoporosis, diabetes) more frequently exhibited fear as the dominant emotion rather than sadness. Axial involvement in PsA is associated with greater disease burden and functional impairment [31–34], which may explain the prevalence of fear-based responses driven by this disease domain. Similarly, comorbid conditions likely amplify concerns about physical health and treatment complications, contributing to heightened fear; in this regard, comorbidities in PsA are well documented to worsen disease severity and quality of life [35–37]. Additionally, these patients also reported significantly more negative valence scores, indicating a worse emotional state.

Hierarchical clustering identified four patient groups with distinct emotional profiles and patterns of disease aspects that patients perceived as most challenging. Valence was more negative in Cluster 1, intermediate in Clusters 2 and 3, and less negative in Cluster 4, with the emotional tone, therefore, varying with the complexity of the patient’s self-reported disease experience.

Across all clusters, fear and sadness emerged as the dominant emotions, although their relative levels differed. Patients in Cluster 1 more often described arthritis, enthesitis, chronic pain, and fatigue as the reported challenging disease aspects. They showed the most negative valence and the highest arousal levels, pointing to a state of emotional tension consistent with the effort required to cope with multiple active symptoms. Cluster 2, largely composed of patients reporting axial involvement as challenging, showed intermediate valence and arousal but the highest disgust scores, which might reflect frustration related to physical limitation and stiffness. Cluster 3, where skin and nail diseases were more frequently reported as the challenging aspects, also displayed a moderately negative valence along with similar fear levels, consistent with the psychosocial burden of visible disease and its impact on self-image. Clusters 1, 2, and 3 all showed higher levels of fear, suggesting the predominance of more active negative emotions in patients facing demanding physical or social aspects of PsA. In contrast, Cluster 4, characterized by fewer reported challenges but a high prevalence of fatigue, showed the least negative valence and lowest arousal, with reduced fear but higher sadness. This pattern points to possibly more passive emotional states rather than acute distress linked to fear.

Although this cross-sectional analysis does not allow causal conclusions, the observed patterns underline that emotional responses in difficult-to-treat PsA might vary according to the aspects of the disease patients find most burdensome. Considering these emotional dimensions may help to provide a broader understanding of the patient experience beyond clinical activity alone.

Several limitations should be acknowledged. First, the survey was specifically designed to explore patients’ perspectives on treatment inefficacies and the concepts of C2M-PsA and TR-PsA, which may have biased the emotional tone. Second, the cross-sectional design precludes causal inferences regarding the relationship between disease manifestations and emotional patterns. It remains unclear whether the negative emotional states identified reflect a direct response to treatment failure and disease burden, or whether pre-existing emotional profiles contribute to the perception of the disease as refractory or complex-to-manage—a bidirectional relationship that longitudinal studies will be needed to disentangle. Third, the sentiment analysis methodology required a substantial amount of textual data per respondent to ensure reliability. Consequently, only patients who completed all three core questions were included in the emotional profiling analyses, which may have introduced selection bias. As the survey was voluntary and anonymous, no follow-up was possible to retrieve missing data. Descriptive comparison with the full cohort revealed sociodemographic differences, particularly a higher proportion of females and more highly educated respondents in the analyzed subset, suggesting a possible response propensity bias. These differences should be considered when interpreting the generalizability of findings. Furthermore, cultural background and gender are known to influence emotional expression and illness perception in chronic diseases, with women generally reporting higher emotional expressivity and certain cultural groups showing distinct patterns of somatic versus affective symptom reporting. The overrepresentation of white females in this cohort may therefore have shaped the emotional profiles identified, and caution is warranted in extrapolating these findings to more diverse populations.

From a technical point of view, the use of a relatively compact model (Meta Llama 3.2 3B Instruct) may have constrained performance on shorter or more ambiguous responses; future studies should evaluate whether larger parameter models yield incremental gains in sentiment classification accuracy within similar clinical contexts. A formal stability check—re-running the model on identical narratives across multiple iterations—was not performed. Although the temperature parameter was set to 0.3 to minimize stochastic variability and promote response consistency, residual run-to-run variation cannot be fully excluded. Additionally, although responses in languages other than English were translated into English prior to LLM processing, automated translation may have introduced subtle distortions in emotional tone or nuance that could affect sentiment classification accuracy.

While Ekman’s framework includes more negative than positive emotions, this framework aligns with our results and facilitates the identification of distinct affective profiles. Interestingly, sadness and fear may represent qualitatively different negative states, suggesting potential for targeted interventions. The use of a locally-deployed LLM enabled standardized analysis of qualitative responses and minimized interpretative bias. Additionally, the cluster analysis yielded insights on how emotional experience varies by disease phenotype, underlining the need for personalized care.

In conclusion, this study demonstrates the utility of NLP and LLM-based sentiment analysis for exploring emotional expression in PsA. Predominantly negative emotions, particularly sadness and fear, were identified and associated with higher disease burden. These findings underscore the importance of integrating emotional and psychosocial insights into PsA management to complement clinical assessments. Future longitudinal studies should explore how emotional profiles evolve over time and how they respond to treatment. LLM-guided sentiment analysis offers a promising adjunct to traditional assessment tools, though it should complement—not replace—comprehensive psychological evaluation.

## Supplementary Information

Below is the link to the electronic supplementary material.

Supplementary file1 (DOCX 41 KB)

Supplementary file2 (DOCX 19 KB)

## Data Availability

Data are available upon reasonable request to the corresponding author.
