# Supplementary material for: Local LLM-based sentiment analysis of emotional patterns in complex-to-manage and treatment-resistant psoriatic arthritis
Source: Intern Emerg Med. 2026 Jun 7;21(6):2121–32. doi: 10.1007/s11739-026-04396-w (PMC13582069; doi:10.1007/s11739-026-04396-w)
Supplement: Supplementary file 1 [file 11739_2026_4396_MOESM1_ESM.docx]

|  | Total cohort (N=570) | Analyzed subset  (N=234) |
| --- | --- | --- |
| **Age** |  |  |
| <30 years | 30 (5.3) | 14 (6.0) |
| 30-39 years | 74 (12.9) | 26 (11.1) |
| 40-49 years | 126 (22.1) | 46 (19.7) |
| 50-59 years | 184 (32.3) | 79 (33.8) |
| ≥ 60 years | 156 (27.4) | 69 (29.5) |
| **Gender** |  |  |
| Male | 178 (31.2) | 59 (25.2) |
| Female | 392 (68.8) | 175 (74.8) |
| **Ethnicity** |  |  |
| Asian | 73 (12.8) | 22 (9.4) |
| Black | 8 (1.4) | 2 (0.9) |
| Hispanic | 59 (10.4) | 23 (9.8) |
| White | 414 (72.6) | 181 (77.4) |
| Other | 16 (2.8) | 6 (2.6) |
| **Marital Status** |  |  |
| Single | 116 (20.4) | 44 (18.8) |
| Married/Living with partner | 374 (65.6) | 153 (65.4) |
| Divorced/Separated | 54 (9.5) | 23 (9.8) |
| Widowed | 26 (4.6) | 14 (6.0) |
| **Education Level** |  |  |
| Elementary/Primary school | 43 (7.5) | 20 (8.5) |
| High school/Secondary school | 374 (65.6) | 55 (23.5) |
| College degree | 54 (9.5) | 106 (45.3) |
| Postgraduate degree | 26 (4.6) | 53 (22.6) |
| **Occupational Status** |  |  |
| Homemaker | 39 (6.8) | 17 (7.3) |
| Unemployed | 36 (6.3) | 15 (6.4) |
| Student | 5 (0.9) | 1 (0.4) |
| Employed part-time | 102 (17.9) | 44 (18.8) |
| Employed full-time | 204 (35.8) | 79 (33.8) |
| On leave of absence | 21 (3.7) | 6 (2.6) |
| On disability | 47 (8.2) | 21 (9.0) |
| Retired | 116 (20.4) | 51 (21.8) |

**Supplementary Tab.1** Sociodemographic characteristics of the psoriatic arthritis patients who answered the survey (n.570) and analzysed subset (N=234). The latter comprises patients with complete sentiment analysis data. Education level categories in the analyzed subset diverge proportionally from the total cohort, likely reflecting differential response rates across education strata.

| Challenging disease aspects | Total cohort (N=570) | Analyzed subset  (N=234) |  |
| --- | --- | --- | --- |
| Arthritis | 384 (67.37%) | 171 (73.1%) |  |
| Enthesitis | 269 (47.19%) | 130 (55.6%) |  |
| Dactylitis | 189 (33.16%) | 87 (37.2%) |  |
| Skin psoriasis | 286 (50.18%) | 118 (50.4%) |  |
| Nail involvement | 183 (32.11%) | 79 (33.8%) |  |
| Axial disease | 287 (50.35%) | 139 (59.4%) |  |
| Uveitis | 66 (11.58%) | 35 (15.0%) |  |
| Inflammatory bowel disease | 73 (12.81%) | 28 (12.0%) |  |
| Depression/anxiety | 175 (30.70%) | 84 (35.9%) |  |
| Chronic pain | 268 (47.02%) | 130 (55.6%) |  |
| Fatigue | 384 (67.37%) | 181 (77.4%) |  |
| Other comorbidities – Obesity, hypertension, osteoporosis, diabetes, etc. | 165 (28.95%) | 83 (35.5%) |  |

**Supplementary Tab.2** Frequency of patient-reported disease aspects that contributed to their condition being perceived as treatment refractory and/or complex-to-manage (Q1-2 of the survey). These features were later used in the correlation analyses with emotional valence and discrete emotions derived from sentiment analysis of open-ended responses.

| **Demographic Variable** | **Group** | **n** | **Valence, Mean ± SD** | **Arousal, Mean ± SD** |
| --- | --- | --- | --- | --- |
| **Gender** |  |  |  |  |
|  | Female | 174 | -0.58 ± 0.25 | 0.64 ± 0.10 |
|  | Male | 60 | -0.53 ± 0.25 | 0.65 ± 0.10 |
|  | p-value |  | 0.29 | 0.66 |
| **Age group** |  |  |  |  |
|  | 18-29 years | 14 | -0.57 ± 0.24 | 0.68 ± 0.07 |
|  | 30-39 years | 26 | -0.49 ± 0.29 | 0.65 ± 0.09 |
|  | 40-49 years | 46 | -0.60 ± 0.18 | 0.65 ± 0.09 |
|  | 50-59 years | 79 | -0.56 ± 0.26 | 0.64 ± 0.09 |
|  | ≥60 years | 69 | -0.57 ± 0.27 | 0.64 ± 0.11 |
|  | p-value |  | 0.51 | 0.62 |
| **Race and ethnicity** |  |  |  |  |
|  | Asian | 22 | -0.66 ± 0.18 | 0.67 ± 0.08 |
|  | Black | 2 | -0.20 ± 0.52 | 0.62 ± 0.02 |
|  | Hispanic | 23 | -0.54 ± 0.22 | 0.63 ± 0.12 |
|  | White | 181 | -0.56 ± 0.25 | 0.65 ± 0.10 |
|  | Other | 6 | -0.53 ± 0.30 | 0.63 ± 0.06 |
|  | p-value |  | 0.09 | 0.66 |
| **Relationship status** |  |  |  |  |
|  | Single | 44 | -0.52 ± 0.29 | 0.65 ± 0.09 |
|  | Married/Partnership | 153 | -0.57 ± 0.24 | 0.65 ± 0.10 |
|  | Divorced/Separated | 23 | -0.66 ± 0.17 | 0.67 ± 0.10 |
|  | Widowed | 14 | -0.47 ± 0.31 | 0.59 ± 0.11 |
|  | p-value |  | 0.07 | 0.16 |
| **Education level** |  |  |  |  |
|  | Elementary school | 20 | -0.65 ± 0.23 | 0.66 ± 0.10 |
|  | High school | 55 | -0.53 ± 0.24 | 0.64 ± 0.09 |
|  | College degree | 106 | -0.58 ± 0.25 | 0.63 ± 0.10 |
|  | Postgraduate degree | 53 | -0.55 ± 0.26 | 0.67 ± 0.09 |
|  | p-value |  | 0.28 | 0.12 |
| **Occupational status** |  |  |  |  |
|  | Homemaker | 17 | -0.52 ± 0.23 | 0.65 ± 0.08 |
|  | Unemployed | 15 | -0.59 ± 0.21 | 0.66 ± 0.08 |
|  | Student | 1 | -0.03 ± — | 0.80 ± — |
|  | Employed part-time | 44 | -0.58 ± 0.23 | 0.65 ± 0.08 |
|  | Employed full-time | 79 | -0.55 ± 0.25 | 0.64 ± 0.10 |
|  | On leave of absence | 6 | -0.46 ± 0.36 | 0.67 ± 0.09 |
|  | On disability | 21 | -0.67 ± 0.18 | 0.65 ± 0.10 |
|  | Retired | 51 | -0.58 ± 0.29 | 0.64 ± 0.11 |
|  | p-value |  | 0.17 | 0.80 |

**Supplementary Tab.3 Descriptive statistics and ANOVA results for emotional dimensions across demographic characteristics in patiens who completed the survey (n=234).** Valence scores range from -1 (most negative) to +1 (most positive), and arousal scores range from 0 (low activation) to 1 (high activation). All p-values were non-significant (p > 0.05), indicating no significant differences in emotional dimensions across demographic groups.

| **Demographic Variable** | **Group** | **n** | **Anger**  **Mean ± SD** | **Disgust**  **Mean ± SD** | **Fear**  **Mean ± SD** | **Joy**  **Mean ± SD** | **Sadness**  **Mean ± SD** | **Surprise**  **Mean ± SD** |
| --- | --- | --- | --- | --- | --- | --- | --- | --- |
| **Gender** |  |  |  |  |  |  |  |  |
|  | Male | 60 | 0.56 ± 1.87 | 14.28 ± 7.46 | 32.65 ± 12.27 | 2.38 ± 1.97 | 40.43 ± 16.15 | 9.72 ± 4.98 |
|  | Female | 174 | 0.96 ± 2.93 | 14.37 ± 6.45 | 32.76 ± 11.06 | 3.17 ± 3.42 | 38.12 ± 13.52 | 10.63 ± 4.70 |
|  | p-value |  | 0.22 | 0.94 | 0.95 | 0.03* | 0.33 | 0.22 |
| **Age group** |  |  |  |  |  |  |  |  |
|  | 18-29 years | 14 | 0.95 ± 3.56 | 15.01 ± 9.83 | 28.21 ± 9.97 | 1.63 ± 1.56 | 43.75 ± 15.20 | 10.46 ± 3.94 |
|  | 30-39 years | 26 | 1.09 ± 2.69 | 15.07 ± 7.46 | 34.59 ± 11.41 | 2.99 ± 2.06 | 36.63 ± 15.50 | 9.64 ± 5.75 |
|  | 40-49 years | 46 | 0.70 ± 2.48 | 14.82 ± 6.14 | 34.04 ± 11.11 | 2.65 ± 2.90 | 36.59 ± 14.37 | 11.22 ± 4.67 |
|  | 50-59 years | 79 | 1.14 ± 3.20 | 13.76 ± 5.83 | 31.50 ± 11.26 | 3.65 ± 4.19 | 39.58 ± 14.26 | 10.37 ± 4.74 |
|  | ≥60 years | 69 | 0.52 ± 1.94 | 14.31 ± 7.10 | 33.50 ± 11.77 | 2.67 ± 2.21 | 38.85 ± 13.42 | 10.16 ± 4.71 |
|  | p-value |  | 0.68 | 0.87 | 0.31 | 0.11 | 0.46 | 0.70 |
| **Race and ethnicity** |  |  |  |  |  |  |  |  |
|  | Asian | 22 | 0.81 ± 2.09 | 14.93 ± 9.34 | 33.43 ± 9.34 | 1.22 ± 1.24 | 38.92 ± 15.31 | 10.70 ± 4.92 |
|  | Black | 2 | 0.00 ± 0.00 | 11.40 ± 7.50 | 34.45 ± 21.99 | 4.73 ± 4.71 | 34.58 ± 31.23 | 14.85 ± 6.48 |
|  | Hispanic | 23 | 0.53 ± 1.77 | 13.79 ± 3.90 | 30.09 ± 11.96 | 3.62 ± 5.54 | 41.47 ± 14.84 | 10.52 ± 4.42 |
|  | White | 181 | 0.94 ± 2.91 | 14.38 ± 6.69 | 32.75 ± 11.54 | 3.07 ± 2.87 | 38.52 ± 14.11 | 10.35 ± 4.86 |
|  | Other | 6 | 0.00 ± 0.00 | 14.26 ± 5.57 | 39.14 ± 5.31 | 3.49 ± 0.77 | 34.19 ± 6.62 | 8.93 ± 2.50 |
|  | p-value |  | 0.86 | 0.95 | 0.51 | 0.07 | 0.80 | 0.66 |
| **Relationship status** |  |  |  |  |  |  |  |  |
|  | Single | 44 | 1.12 ± 2.81 | 14.04 ± 6.81 | 31.89 ± 12.38 | 2.97 ± 2.89 | 40.97 ± 16.08 | 9.01 ± 4.62 |
|  | Married/Partnership | 153 | 0.89 ± 2.84 | 14.52 ± 6.91 | 32.84 ± 11.04 | 3.13 ± 3.41 | 38.14 ± 13.86 | 10.50 ± 4.80 |
|  | Divorced/Separated | 23 | 0.68 ± 2.34 | 14.24 ± 5.00 | 35.01 ± 10.27 | 1.44 ± 1.44 | 35.95 ± 13.31 | 12.70 ± 4.59 |
|  | Widowed | 14 | 0.00 ± 0.00 | 13.60 ± 7.04 | 30.40 ± 13.45 | 3.82 ± 1.96 | 42.25 ± 13.32 | 9.93 ± 4.22 |
|  | p-value |  | 0.59 | 0.95 | 0.62 | 0.07 | 0.38 | 0.03* |
| **Education level** |  |  |  |  |  |  |  |  |
|  | Elementary school | 20 | 1.38 ± 4.99 | 13.31 ± 7.34 | 33.24 ± 14.12 | 3.15 ± 5.77 | 38.41 ± 15.82 | 10.53 ± 4.40 |
|  | High school | 55 | 0.42 ± 1.54 | 14.67 ± 6.53 | 32.28 ± 12.04 | 2.81 ± 1.93 | 39.27 ± 15.06 | 10.57 ± 4.80 |
|  | College degree | 106 | 0.82 ± 2.65 | 14.82 ± 7.22 | 33.65 ± 10.27 | 2.82 ± 2.59 | 37.81 ± 13.45 | 10.10 ± 4.83 |
|  | Postgraduate degree | 53 | 1.18 ± 2.48 | 13.47 ± 5.47 | 31.16 ± 11.62 | 3.39 ± 3.73 | 40.02 ± 14.48 | 10.78 ± 4.88 |
|  | p-value |  | 0.40 | 0.56 | 0.61 | 0.70 | 0.81 | 0.84 |
| **Occupational status** |  |  |  |  |  |  |  |  |
|  | Homemaker | 17 | 0.98 ± 2.29 | 14.98 ± 5.46 | 34.91 ± 11.55 | 4.88 ± 5.96 | 32.79 ± 9.26 | 11.46 ± 4.70 |
|  | Unemployed | 15 | 0.00 ± 0.00 | 12.93 ± 8.14 | 29.94 ± 11.39 | 1.22 ± 1.35 | 44.04 ± 17.42 | 11.89 ± 4.06 |
|  | Student | 1 | 0.00 ± — | 10.00 ± — | 23.33 ± — | 3.33 ± — | 55.00 ± — | 8.33 ± — |
|  | Employed part-time | 44 | 1.88 ± 4.35 | 13.73 ± 6.73 | 31.34 ± 11.31 | 3.75 ± 3.89 | 39.94 ± 13.39 | 9.37 ± 4.27 |
|  | Employed full-time | 79 | 0.49 ± 2.06 | 14.60 ± 6.59 | 33.07 ± 11.08 | 2.88 ± 2.73 | 38.30 ± 15.23 | 10.67 ± 5.06 |
|  | On leave of absence | 6 | 1.86 ± 2.87 | 12.09 ± 4.20 | 29.44 ± 6.98 | 3.66 ± 1.29 | 43.06 ± 10.39 | 9.90 ± 2.33 |
|  | On disability | 21 | 1.08 ± 2.75 | 15.42 ± 5.85 | 34.80 ± 11.55 | 2.76 ± 1.88 | 36.44 ± 15.46 | 9.49 ± 4.97 |
|  | Retired | 51 | 0.57 ± 2.02 | 14.58 ± 7.50 | 33.22 ± 12.29 | 2.32 ± 2.15 | 38.76 ± 13.26 | 10.56 ± 5.11 |
|  | p-value |  | 0.14 | 0.89 | 0.77 | 0.02* | 0.35 | 0.59 |

**Supplementary Tab. 4 Descriptive statistics and ANOVA results for basic emotions across demographic characteristics.** Values represent mean scores for Ekman's six basic emotions (anger, disgust, fear, joy, sadness, surprise). Significant differences were found only for: joy by gender (p = 0.03) and occupational status (p=0.02), surprise by relationship status (p = 0.03). All other comparisons were non-significant (p > 0.05). * indicates p < 0.05.
