# Supplementary material for: Local LLM-based sentiment analysis of emotional patterns in complex-to-manage and treatment-resistant psoriatic arthritis
Source: Intern Emerg Med. 2026 Jun 7;21(6):2121–32. doi: 10.1007/s11739-026-04396-w (PMC13582069; doi:10.1007/s11739-026-04396-w)
Supplement: Supplementary file 2 [file 11739_2026_4396_MOESM2_ESM.docx]

**PROMPT STRATEGY**

```

testo (…)

from openai import OpenAI
import pandas as pd
import numpy as np


client = OpenAI(base_url="http://localhost:1234/v1", api_key="not-needed")


prompt = """[INST] You are an expert in emotion analysis. Analyze the following text using only Paul Ekman's six basic emotions.

**CRITICAL**: Provide an exact percentage breakdown for each emotion. The six percentages MUST sum to exactly 100%.

**ANGER**: Direct rage, blame, or criticism toward specific people, world or systems
Examples: "The doctors don't listen to me", "The system failed me", "My rheumatologist is incompetent"

**SADNESS**: Feeling defeated, powerless, hopeless, or overwhelmed by circumstances
Examples: "I can't function", "I'm overwhelmed", "I can't cope anymore", "I'm exhausted"

**FEAR**: Anxiety, worry, or concern about future outcomes or consequences
Examples: "I'm scared it will get worse", "What if treatments stop working", "I'm afraid of side effects"

**DISGUST**: Physical revulsion, rejection, or strong aversion to symptoms or treatments
Examples: "The medication makes me sick", "I hate these treatments", "The side effects are disgusting"

**JOY**: Positive emotions including happiness, relief, gratitude, or hope
Examples: "I'm grateful for my treatment", "Finally some relief", "I feel hopeful"

**SURPRISE**: Shock, unexpected reactions, or sudden realizations
Examples: "I was shocked by the diagnosis", "I didn't expect this reaction", "Suddenly everything changed"

Please respond only in the following format:
EMOTIONS: anger|disgust|fear|joy|sadness|surprise
Example:
EMOTIONS: 5|0|15|0|75|5

Now, analyze this text: [/INST]"""

	# Normalization
def normalize_emotions(emotion_dict):
    total = sum(emotion_dict.values())
    if total > 0:
        return {key: round((value/total)*100, 1) for key, value in emotion_dict.items()}
    else:
        return {key: round(100/6, 1) for key in emotion_dict.keys()}

results = []

for response in testo:
    result = {
        'text': response,
        'anger': np.nan,
        'disgust': np.nan,
        'fear': np.nan,
        'joy': np.nan,
        'sadness': np.nan,
        'surprise': np.nan
    }

    if len(response.strip()) > 0:
        try:
            response_llm = client.chat.completions.create(
                model="llama-3.2-3b-instruct",
                messages=[{"role": "user", "content": prompt + response}],
                temperature=0.3
            )

            output = response_llm.choices[0].message.content.strip()
            line = output.split('EMOTIONS: ')[1]
            emotions = line.split('|')
            emotion_names = ['anger', 'disgust', 'fear', 'joy', 'sadness', 'surprise']
            emotion_props = {name: float(value) for name, value in zip(emotion_names, emotions)}

            # Normalization
            emotion_props = normalize_emotions(emotion_props)

            result.update(emotion_props)

            # Print the sum for checking
            total_sum = sum(emotion_props.values())
            print(f"Somma: {total_sum}")

        except Exception as e:
            print(f"Errore nell'analisi: {e}")

    results.append(result)

df = pd.DataFrame(results)

emotion_cols = ['anger', 'disgust', 'fear', 'joy', 'sadness', 'surprise']
df['total_check'] = df[emotion_cols].sum(axis=1)

pd.set_option('display.max_rows', None)
pd.set_option('display.max_columns', None)
pd.set_option('display.width', None)
pd.set_option('display.max_colwidth', None)

print("\nDataFrame completo:")
print(df)

print("\nStatistiche di riepilogo:")
print(df[emotion_cols].describe())

print(f"\nNumero totale di risposte analizzate: {len(df)}")

print(f"\nValori unici delle somme: {df['total_check'].dropna().unique()}")

df.to_excel("Ekman_Sentiment_OnlyQ1.xlsx", index=False)
```

from openai import OpenAI
import pandas as pd
import numpy as np

existing_df = pd.read_excel("Ekman_Sentiment_OnlyQ1.xlsx")

client = OpenAI(base_url="http://localhost:1234/v1", api_key="not-needed")

dimensions_prompt = """[INST] You are an emotion analysis expert. Analyze the emotional dimensions of the following text.

You must provide:
1. Valence (from -1 to +1): how positive or negative the emotion is
2. Arousal (from 0 to 1): level of physiological activation (NEVER negative!)

**Quick Examples:**
- High negative valence + low arousal: "I'm exhausted and hopeless" → DIMENSIONS: -0.8|0.2
- High negative valence + high arousal: "I'm furious about this!" → DIMENSIONS: -0.7|0.9
- Low negative valence + medium arousal: "I'm somewhat worried" → DIMENSIONS: -0.3|0.5

**CRITICAL: Arousal must be from 0.0 to 1.0 (never negative!)**

Respond ONLY in this format:
DIMENSIONS: valence|arousal

Example:
DIMENSIONS: -0.8|0.7

Analyze this text: [/INST]"""


dimensions_results = []

for i, response in enumerate(testo):
 print(f"Analizzando dimensioni {i+1}/{len(testo)}: {response[:50] if response else 'EMPTY'}...")

 result = {
 'text': response,
 'valence': np.nan,
 'arousal': np.nan
 }

 if len(response.strip()) > 0:
 try:
 dimensions_response = client.chat.completions.create(
 model="llama-3.2-3b-instruct",
 messages=[{"role": "user", "content": dimensions_prompt + response}],
 temperature=0.3
 )

 dimensions_output = dimensions_response.choices[0].message.content.strip()
 dimensions_line = dimensions_output.split('DIMENSIONS: ')[1]
 valence, arousal = dimensions_line.split('|')
 result['valence'] = float(valence)
 result['arousal'] = float(arousal)

 print(f" ✅ Valence: {result['valence']}, Arousal: {result['arousal']}")

 except Exception as e:
 print(f" ❌ Errore: {e}")
 else:
 print(f" ⚪ Testo vuoto")

 dimensions_results.append(result)

dimensions_df = pd.DataFrame(dimensions_results)

*# Combine df*
final_df = pd.concat([existing_df, dimensions_df[['valence', 'arousal']]], axis=1)

print("\n=== RISULTATI DIMENSIONI ===")
print(f"Analisi completate: {len(dimensions_df.dropna(subset=['valence', 'arousal']))}")
print("\nStatistiche:")
print(dimensions_df[['valence', 'arousal']].describe())

*# Save dimensions*
dimensions_df.to_excel("Valence_Arousal_Only.xlsx", index=False)
print("\nFile salvato: Valence_Arousal_Only.xlsx")

```
# Combine DF to obtain a single DF for analysis
emotions_df = pd.read_excel('Ekman_Sentiment_OnlyQ1.xlsx')
dimensions_df = pd.read_excel('Valence_Arousal_Only.xlsx')
combined_df = pd.concat([emotions_df, dimensions_df[['valence', 'arousal']]], axis=1)
combined_df.to_excel('Combined_Emotions_Dimensions_q1.xlsx', index=False)
```
